# Chronic Pelvic Peritonitis

**Published:** 1894-09

**Authors:** 


CHRONIC PELVIC PERITONITIS.—M. M., single; age twenty-three; occupation, servant; was admitted April 23, 1894. Family history: Father living and in good health; mother died thirteen years since, cause unknown.

Individual history: Patient was never sick before. Gave birth to a child two years before. Habits temperate.

Present illness: Contracted a cold about two months ago, followed by severe cough, pain in abdomen and severe headache. When admitted into hospital she assumed constantly the dorsal position; countenance apathetic and indifferent; skin clean; appetite lost; bowels regular; tongue coated; urine highly colored and causes burning in passage. Had not menstruated for two months. Has constant headache.

Physical examination: Pulse 100; respiration 24; temperature normal. General tenderness over abdomen, lessened by flexion of thighs. No vaginal trouble. Uterus adherent—fixed.

Treatment: Hot poultices over abdomen and Dover's powders to relieve tenderness and pain, and given the following reconstructive tonic:

℞ Codliver glycerine.......................................℥ viij.
Nux vom. tr..............................................ʒ ij.
M. Sig.—Teaspoonful every four or five hours.

Patient was discharged much improved.

